# DSS and DHS: longitudinal and cross-sectional viewpoints on child and adolescent mortality in Ethiopia

**DOI:** 10.1186/1478-7954-5-12

**Published:** 2007-12-27

**Authors:** Peter Byass, Alemayehu Worku, Anders Emmelin, Yemane Berhane

**Affiliations:** 1 Umeå International School of Public Health, Umeå University, Umeå, Sweden; 2 Department of Community Health, Addis Ababa University, Addis Ababa, Ethiopia; 3 Addis Continental Institute of Public Health, PO Box 26751/1000, Addis Ababa, Ethiopia

## Abstract

**Background:**

In countries where routine vital registration data are scarce, Demographic Surveillance Sites (DSS: locally defined populations under longitudinal surveillance for vital events and other characteristics) and Demographic and Health Surveys (DHS: periodic national cluster samples responding to cross-sectional surveys) have become standard approaches for gathering at least some data. This paper aims to compare DSS and DHS approaches, seeing how they complement each other in the specific instance of child and adolescent mortality in Ethiopia.

**Methods:**

Data from the Butajira DSS 1987–2004 and the Ethiopia DHS rounds for 2000 and 2005 formed the basis of comparative analyses of mortality rates among those aged under 20 years, using Poisson regression models for adjusted rate ratios.

**Results:**

Patterns of mortality over time were broadly comparable using DSS and DHS approaches. DSS data were more susceptible to local epidemic variations, while DHS data tended to smooth out local variation, and be more subject to recall bias.

**Conclusion:**

Both DSS and DHS approaches to mortality surveillance gave similar overall results, but both showed method-dependent advantages and disadvantages. In many settings, this kind of joint-source data analysis could offer significant added value to results.

## Introduction

The acute shortage of reliable population-based health data in the world's poorer countries, where complete and compulsory registration of vital events and other health data is currently impracticable or unaffordable, have led to other strategies for obtaining at least some data. Two major approaches in current use are Demographic Surveillance Sites (DSS) and Demographic and Health Surveys (DHS).

A number of DSSs have been established, mainly across Africa and Asia, the majority of which are affiliated to the Indepth Network [1]. Although there are local and contextual variations in the design of individual DSSs, which generally operate autonomously, the basic concept involves the identification of a geographically defined population, typically of 30 to 100 thousand people. An initial census of the defined population forms the basis for an open cohort that can be followed longitudinally. New individuals can enter the cohort by birth or in-migration, while cohort members can exit by out-migration or death. Alongside surveillance of vital events (birth, migration and death), other health-relevant parameters such as household characteristics, individual characteristics and experiences of health and disease are routinely collected. Established DSSs can also offer unique opportunities as platforms for interventions, such as vaccine trials.

The DHS programme is a global U.S. Government-backed initiative to undertake relatively large and complex cross-sectional surveys of demographic and health parameters on nationally representative samples in poorer and middle-income countries [2]. The underlying methodology for this has been standardised, apart from small details relating to country-specific requirements, and both reports and datasets from particular DHS rounds are made available via the internet. Nationally distributed cluster samples of households are taken as the basis for a DHS round. DHSs are repeated at approximately 5-year intervals in many countries around the world, with each round drawing a fresh cross-sectional sample.

In Ethiopia, the Butajira Rural Health Programme (BRHP) is a DSS that has been in place since 1987 and has accumulated over 700,000 person-years of surveillance data. It is located in the Southern Nations, Nationalities and Peoples Region (SNNPR), some 130 km to the south of Addis Ababa. This district was purposefully chosen as being potentially representative for a DSS in 1986, on the basis of being at least 100 km from any major city, but not in a peripheral border region; combining a mixture of the highland and lowland environments typical of Ethiopia; and containing a mixture of ethnic and religious groups. National DHS sample surveys have been undertaken in Ethiopia in 2000 and 2005.

The aim of this paper is to review child and adolescent mortality outputs from both the BRHP DSS and the Ethiopian DHS data from the 2000 and 2005 rounds, comparing findings within areas of common ground in an attempt to see how the two different approaches complement and speak to each other.

## Methods

The BRHP DSS dataset started with an initial enumerated cohort of 28,580 persons resident in 10 communities (9 rural, 1 urban) in the Butajira District on 1^st ^January 1987. The sampled communities were selected from the entire district using a probability proportional to size approach and covered approximately 10% of the district. All of the households have since been visited regularly to record vital events (births, deaths, in and out migrations) and the dataset describing this dynamic cohort up to 31^st ^December 2004 was used for this analysis. By this time the resident population had grown to 54,419 [3].

From this dataset, records relating to all individuals who spent any time between birth and their twentieth birthday as residents in the BRHP cohort were extracted, and compiled into a format that included person-time of exposure according to age group (under 1 year, 1 to 4 years, 5 years and over), sex and urban/rural residence. A total of 68,979 individuals were included, each contributing an average of 6.0 person-years. Deaths were recorded for 6,345 individuals.

The DHS 2000 and 2005 datasets for Ethiopia both included detailed birth histories taken from women in the household sample, which included date of birth for ever-born children and date of death for those no longer alive at the time of interview. In 2000, interviews were completed for 15,367 women aged 15 to 49 years, and in 2005 14,070 women were interviewed [4,5]. The date when women took up residence in their current household was recorded, as well as the interview date. Thus it was possible to construct a pseudo-cohort dataset with a record for each child, covering the same period (1987 to 2004) and same age range (0 to 19 years) as the BRHP dataset. Individual child records from the 2000 and 2005 DHS surveys were allowed to overlap in time, given that the two samples were drawn independently. Person-time for each child started at the latest of: 1^st ^January 1987, date of birth or date of mother's residence starting. Person-time ended at the earliest of: date of death, date of interview, twentieth birthday or 31^st ^December 2004. Variables within the dataset were the same as those extracted in the BRHP DSS dataset. A total of 75,443 children were included, each contributing an average of 7.2 person-years. Deaths were recorded for 10,517 individuals.

Both the DSS and DHS datasets were incorporated into Poisson regression models (using Stata software) in which individual person-time was the exposure variable and death the outcome measure.

## Results

The BRHP DSS dataset included 6,345 deaths among 413,965 person-years in the age range 0 to 19 years during the period 1987 to 2004, a crude mortality rate of 15.3 per 1,000 person-years. The corresponding national DHS dataset included 10,517 deaths among 544,598 person-years, a crude rate of 19.3 per 1,000 person-years. Within these national data, the subset for SNNPR amounted to 1,848 deaths in 84,058 person-years, a crude rate of 22.0 per 1,000 person-years. Table 1 shows a breakdown of deaths and person-time by age group, sex, residence, year and region for both DSS and DHS data.

**Table 1:** Deaths and person-time observed for BRHP DSS (6,345 deaths among 413,965 person-years aged under 20) and Ethiopian DHS data (10,517 deaths among 544,598 person-years aged under 20).

		**BRHP DSS**		**Ethiopia DHS**	
		
factor	level	deaths	person-years (000s)	deaths	person-years (000s)
age group	< 1 year	2,328	24.8	5,227	49.0
	1 to 4 years	2,242	89.9	3,349	170.0
	5 to 19 years	1,775	299.3	1,941	325.6
sex	female	3,016	207.9	4,785	264.3
	male	3,329	206.1	5,732	280.3
residence	rural	5,812	330.2	9,431	439.9
	urban	533	83.7	1,086	104.7
year	1987	202	16.4	576	17.1
	1988	480	17.5	554	19.2
	1989	391	18.6	636	21.1
	1990	356	19.8	677	23.5
	1991	518	20.5	698	26.2
	1992	248	20.8	783	28.8
	1993	352	21.2	760	31.5
	1994	388	21.7	795	34.0
	1995	245	21.6	715	36.7
	1996	259	22.1	676	39.5
	1997	351	23.2	710	42.6
	1998	540	25.1	736	45.6
	1999	844	25.8	746	48.6
	2000	274	26.9	403	28.2
	2001	208	27.4	285	23.4
	2002	237	27.9	262	24.9
	2003	254	28.3	246	26.2
	2004	198	28.4	259	27.6
region	Tigray	-	-	976	53.2
	Affar	-	-	751	31.4
	Amhara	-	-	1,686	84.7
	Oromiya	-	-	1,977	98.9
	Somali	-	-	550	36.7
	Ben-Gumuz	-	-	839	35.1
	SNNP	-	-	1,848	84.1
	Gambela	-	-	597	24.4
	Harari	-	-	497	27.2
	Addis Ababa	-	-	283	42.0
	Dire Dawa	-	-	513	27.0

A Poisson regression model of mortality rate ratios was built, including year, age group (under 1 year, 1 to 4 years and 5 to 19 years), sex, urban/rural residence and, for the national DHS data, region. Mortality rate ratios for the BRHP DSS and national DHS data, adjusted for age group, sex, urban/rural residence and, in the case of DHS, for region, are shown in Table 2.

**Table 2:** Adjusted mortality rate ratios for BRHP DSS (6,345 deaths among 413,965 person-years aged under 20) and Ethiopia DHS data (10,517 deaths among 544,598 person-years aged under 20).

		**BRHP DSS**		**Ethiopia DHS**	
		
factor	level	adjusted rate ratio	95% CI	adjusted rate ratio	95% CI
age group	< 1 year	1.0	(ref)	1.0	(ref)
	1 to 4 years	0.271	0.255 to 0.287	0.190	0.182 to 0.199
	5 to 19 years	0.067	0.062 to 0.071	0.062	0.059 to 0.065
sex	female	1.0	(ref)	1.0	(ref)
	male	1.090	1.037 to 1.146	1.138	1.095 to 1.184
residence	rural	1.0	(ref)	1.0	(ref)
	urban	0.395	0.362 to 0.432	0.665	0.614 to 0.720
year	1987	1.0	(ref)	1.0	(ref)
	1988	2.133	1.809 to 2.516	0.897	0.797 to 1.009
	1989	1.780	1.500 to 2.112	1.019	0.909 to 1.042
	1990	1.439	1.211 to 1.711	0.951	0.850 to 1.064
	1991	2.043	1.735 to 2.405	0.882	0.789 to 0.986
	1992	1.032	0.857 to 1.243	0.929	0.833 to 1.036
	1993	1.447	1.216 to 1.721	0.845	0.758 to 0.944
	1994	1.506	1.270 to 1.786	0.847	0.760 to 0.944
	1995	1.002	0.831 to 1.208	0.716	0.641 to 0.801
	1996	1.017	0.846 to 1.223	0.642	0.573 to 0.718
	1997	1.242	1.044 to 1.477	0.620	0.555 to 0.693
	1998	1.815	1.544 to 2.134	0.620	0.555 to 0.692
	1999	2.920	2.504 to 3.406	0.603	0.540 to 0.674
	2000	0.963	0.803 to 1.156	0.581	0.511 to 0.661
	2001	0.685	0.564 to 0.832	0.479	0.415 to 0.553
	2002	0.776	0.643 to 0.936	0.428	0.369 to 0.496
	2003	0.886	0.736 to 1.067	0.407	0.350 to 0.473
	2004	0.691	0.567 to 0.841	0.418	0.360 to 0.484
region	Tigray	-	-	1.0	(ref)
	Affar	-	-	1.287	1.168 to 1.419
	Amhara	-	-	1.119	1.032 to 1.213
	Oromiya	-	-	1.100	1.017 to 1.189
	Somali	-	-	0.817	0.735 to 0.909
	Ben-Gumuz	-	-	1.274	1.160 to 1.400
	SNNP	-	-	1.195	1.104 to 1.294
	Gambela	-	-	1.357	1.224 to 1.505
	Harari	-	-	1.170	1.045 to 1.310
	Addis Ababa	-	-	0.669	0.574 to 0.779
	Dire Dawa	-	-	1.286	1.148 to 1.441

**Figure 1:**
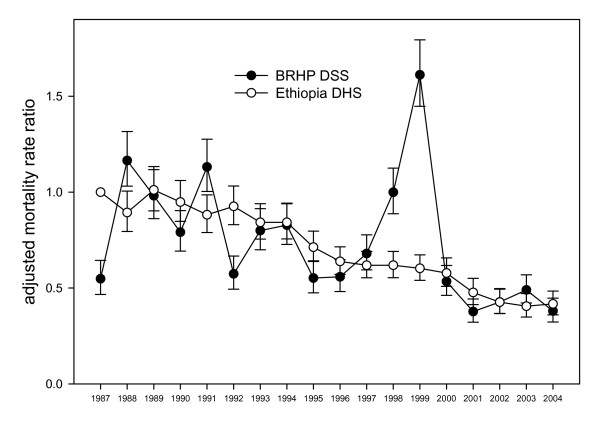
Adjusted mortality rate ratios (aged under 20, adjusted for age group, sex, and urban/rural residence) by year for BRHP DSS data and Ethiopian DHS data, taking DHS data for 1987 (mortality rate 33.7/1,000 person-years) as the reference group. Vertical bars indicate 95% confidence intervals.

Changes in adjusted mortality rate ratios over time for the BRHP DSS data and the national DHS data are shown in Figure 1, taking DHS data for 1987 as the reference group (mortality rate 33.7 per 1,000 person-years). The overall adjusted mortality rate ratio for the national DHS data in comparison to BRHP DSS was 0.93 (95% CI 0.90 to 0.96) (adjusted for age-group, sex, urban-rural residence and year), and for the DHS data from the SNNPR region 0.95 (95% CI 0.90 to 1.00).

**Figure 2:**
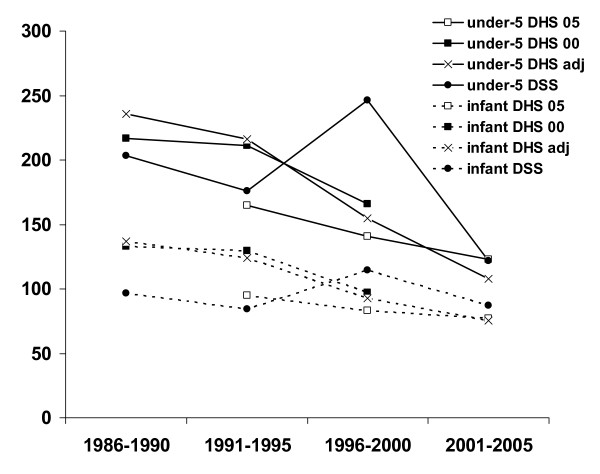
Cumulative under-5 year child mortality and infant mortality rates from Ethiopian 2000 and 2005 DHS and BRHP DSS data, in 5-year periods from DHS survey rounds. DHS 2000 and 2005 figures are taken from DHS reports [4,5]; DHS adjusted figures from a Poisson regression model of combined 2000 and 2005 DHS data; and BRHP DSS results from the same Poisson regression model, adjusted for age-group, sex and urban-rural residence.

Comparisons of the DSS and DHS results for infant and cumulative under-5 year child mortality are shown in Figure 2. DHS figures from the 2000 and 2005 rounds are shown as presented in DHS reports (and thus using standard DHS methods for weighting, etc.) [4-6], compared with adjusted values based on the Poisson regression model described above (combined 2000 and 2005 DHS and the 18-year DSS data). Direct contemporaneous comparisons of DHS mortality shown in Figure 2 between the two survey rounds, with an additional 5 years of recall, suggest that the effects of recall bias may have led to underestimates of infant and under-5 mortality by between 14% and 27% over 5 years.

## Discussion

Overall patterns of child and adolescent mortality as measured longitudinally by the BRHP DSS and cross-sectionally in the 2000 and 2005 DHS rounds in Ethiopia are closely comparable. After adjustment for age group, sex, urban/rural residence and year, the overall rates are within 10% of each other, and the effects of age group and sex are similar. One cannot exclude the possibility that these similarities are entirely coincidental, but, given the purposeful initial selection of Butajira as a district that could be nationally representative, such coincidence is unlikely. Nevertheless, Table 2 reveals many small but statistically significant differences, made possible as a result of the relatively large overall samples.

It is clear that urban or rural residence is an important factor for child mortality, although it is not always a concept that is easy to define. In the BRHP DSS, the "urban" population is one of the four administrative units within Butajira town – and, although this is far from being a large, sophisticated city, the benefits of living in the town compared with adjacent villages have been consistently clear in many analyses from the BRHP DSS [3,7]. It is also clear from the DHS data that urban residence is advantageous – the Addis Ababa region has the lowest regional figures, and in the overall sample urban residents experience significantly lower mortality. The DHS sample was based on census enumeration areas (from the 1994 national census) and each of these areas was officially designated as either urban or rural.

The variation with time is a little more complex, however, and reflects important differences between the two approaches. In 1998–1999, the Butajira area experienced a local drought-related food crisis and appreciable epidemics of infectious disease [3].

Detailed epidemiological evaluations of this are still underway separately, but it seems that abnormal local patterns of rainfall were associated with significant outbreaks of malaria and acute diarrhoea. Lesser cause-specific epidemics were documented in 1998 and 1991 [7]. The geographical extent of these epidemic phenomena beyond the DSS area are unknown, but it is clear that they were not reflected in the national DHS data, nor in the regional figures for SNNPR (data not shown). It remains a moot point as to whether the major epidemics in Butajira were so localised that they should not have been evident in the DHS sample, or whether the retrospective DHS approach just tends to minimise short-term fluctuations. The DSS approach seems to have more scope to detect the extent of local variations in mortality, while the DHS approach has the possible advantage of averaging out local variations across a region, or nationally. These are complementary processes, since both the likely extent of local variation and stable trends over time are both important parameters for health planning. Adequate sample size, in both cases, is important so that confidence intervals can be relatively modest in relation to the extent of variation, as is evident from Figure 1.

Figure 2 illustrates a number of advantages and disadvantages of the DHS and DSS approaches, whilst showing an overall consistency between the two approaches over time. Results from individual DHS rounds appear to under-estimate mortality declines over time, probably due to recall bias effects. Certainly the comparisons of DHS 2000 and DHS 2005 results for the periods 1991–1995 and 1996–2000 show lower figures for the longer-recall sources, and the adjusted analysis of combined DHS data shows a more substantial mortality decline than is seen in either round separately. Since each successive DHS round draws a fresh sample, it is not possible to evaluate the precise source of this bias (for example, missed deaths, missed birth/death pairs, incorrect time estimates for earlier events). This mortality decline is less obvious in the DSS data because of the local epidemic phenomena in 1996–2000.

Variation by region is also an interesting feature of the DHS results, with most inter-regional differences being statistically significant even after adjustment for other factors (Table 1). Ethiopia is a large and diverse country, but it is of interest to see a two-fold range in regional rates, after adjusting for other factors, from a rate ratio of 1.36 (Gambela) to 0.67 (Addis Ababa). If there were more local foci in the sample of sufficient size for separate analysis, one might expect to see a larger range of variation emerging as smaller areas were considered [8]. This is a potential problem in the design of DSS operations, since the inherent need for a DSS often implies that there is little existing data with which to demonstrate that a potential area could be representative. However, these comparisons with national DHS data suggest that the BRHP DSS is nationally representative.

For the purpose of these comparative analyses, the complexities of DSS and DHS sampling design and clustering effects were deliberately ignored, taking individual children as the unit of observation. This had the advantage that exactly the same analytical approach could be applied to the data from both sources, so that the basis of comparison was not confused by methodological differences.

Table 1 reveals some important methodological differences between the DSS and DHS data. The DHS approach tends to over-sample younger children, since younger mothers who are interviewed can only contribute data on younger children. This probably accounts to a large extent for the higher crude mortality observed in the DHS data. In terms of trends over time, the DSS data shows a steady increase in person-time per year, corresponding to the growth of the surveyed population. In contrast, the DHS data yields maximum child person-time at the time of survey rounds, falling off in earlier years. Thus the timing of the survey rounds in 2000 and 2005 is clearly evident from the person-time per year. Trends in numbers of deaths per year are much more stable in the DHS data, not so much because of sample size but due to an averaging over the whole country. The use of multivariate regression modelling was therefore an important part of the comparison process, allowing adjustment for different distributions of key parameters between the DSS and DHS samples.

Although there is now a substantial accumulation of both DSS and DHS datasets from many countries, relatively few comparative analyses have been undertaken. In 1997, an early DHS round for Bangladesh was compared with the Matlab DSS with particular emphasis on fertility rates [9], which concluded that vital rates were comparable within the Matlab area using both approaches, but with some differences. More recently, comparisons of childhood mortality risk factors in Burkina Faso [10] and demographic indices in Mozambique [11] have been published, both concluding that DSS and DHS estimates were reasonably consistent.

Both DSS and DHS operations inevitably suffer from a lack of quality data for validation, since by definition they are implemented in locations where that lack of data is a primary problem. Although this kind of comparison by no means provides a "gold standard" for validating either method, the closely comparable results obtained from two approaches that differ widely conceptually and operationally is encouraging. The extent to which findings from DSSs can reliably and appropriately be extrapolated into wider, surrounding areas is an on-going concern, and an area in which methods are not well established. Conversely, the ability of national sample survey designs as used by DHS to elucidate local patterns and characteristics, as well as trends over time, is dubious. Thus this comparative analysis of the two approaches seems valuable, and could well be undertaken in other countries, and also for parameters other than child mortality, in order to maximise the potential of existing DSS and DHS datasets.

The aim of this paper was not to determine any kind of methodological superiority, but to explore the ways in which two different approaches to measuring child mortality might complement each other. Clearly there are significant and important ways in which the strengths and weaknesses of each method can enhance overall understanding and contribute to essential data for health planning. This kind of joint-source data analysis is to be encouraged.

## Competing interests

The author(s) declare that they have no competing interests.
